# Controlling the dynamics of the Nek2 leucine zipper by engineering of “kinetic” disulphide bonds

**DOI:** 10.1371/journal.pone.0210352

**Published:** 2019-02-01

**Authors:** Daniel S. Gutmans, Sara B-M Whittaker, Karishma Asiani, R. Andrew Atkinson, Alain Oregioni, Mark Pfuhl

**Affiliations:** 1 Randall Centre and School of Cardiovascular Medicine and Sciences, King’s College London, London, United Kingodm; 2 The Henry Wellcome Building for Biomolecular NMR Spectroscopy, Institute of Cancer & Genomic Sciences, University of Birmingham, Birmingham, United Kingodm; 3 Medical Research Council (MRC) Biomolecular NMR Centre, The Francis Crick Institute, London, United Kingdom; Nanyang Technological University, SINGAPORE

## Abstract

Nek2 is a dimeric serine/ threonine protein kinase that belongs to the family of NIMA-related kinases (Neks). Its N-terminal catalytic domain and its C-terminal regulatory region are bridged by a leucine zipper, which plays an important role in the activation of Nek2’s catalytic activity. Unusual conformational dynamics on the intermediary/slow timescale has thwarted all attempts so far to determine the structure of the Nek2 leucine zipper by means of X-ray crystallography and Nuclear Magnetic Resonance (NMR). Disulfide engineering, the strategic placement of non-native disulfide bonds into flexible regions flanking the coiled coil, was used to modulate the conformational exchange dynamics of this important dimerization domain. The resulting reduction in exchange rate leads to substantial improvements of important features in NMR spectra, such as line width, coherence transfer leakage and relaxation. These effects were comprehensively analyzed for the wild type protein, two single disulfide bond-bearing mutants and another double disulfide bonds-carrying mutant. Furthermore, exchange kinetics were measured across a wide temperature range, allowing for a detailed analysis of activation energy (ΔG^‡^) and maximal rate constant (k’_ex_). For one mutant carrying a disulfide bond at its C-terminus, a full backbone NMR assignment could be obtained for both conformers, demonstrating the benefits of the disulfide engineering. Our study demonstrates the first successful application of ‘kinetic’ disulfide bonds for the purpose of controlling the adverse effects of protein dynamics. Firstly, this provides a promising, robust platform for the full structural and functional investigation of the Nek2 leucine zipper in the future. Secondly, this work broadens the toolbox of protein engineering by disulfide bonds through the addition of a kinetic option in addition to the well-established thermodynamic uses of disulfide bonds.

## Introduction

Nek2 is a dimeric serine/threonine protein kinase that belongs to the family of NIMA-related kinases (Neks) [1,2]. Most of its known functions are related to the centrosome and it has been established as an important target for cancer drug development [3,4]. Nek2 comprises an N-terminal catalytic domain and a C-terminal regulatory region, which contains a leucine zipper (LZ) followed immediately by a short coiled coil. The leucine zipper has been shown to play an important in role in the regulation of Nek2 [5].

Earlier structural investigations of the leucine zipper failed because no crystals could be obtained (A. Fry, S. Smerdon, unpublished). Recent NMR studies showed that the leucine zipper exists in two conformations that exchange on a slow time scale (about 17s^−1^) explaining the failure to crystallise [6]. Conformational exchange on this timescale has a significant line broadening effect in NMR spectra thus also challenging NMR spectroscopy as an alternative to structure determination. To select a single conformation and thus remove the exchange process, a disulfide bond had previously been placed into the hydrophobic core, as a result of which the leucine zipper was locked into one conformation (LZ5-K309C/C335A) [6]. Although this approach made the protein amenable to further investigations by NMR, it also brought several disadvantages. Firstly, only one of the two conformers could be generated in this manner [7]. Secondly, while broadly similar spectra were recorded with the disulfide-bearing mutant, its spectra showed considerable deviations in detail from the wildtype spectra which prevented a full assignment of the wild type leucine zipper. Furthermore, NMR samples of LZ5-K309C/C335A lacked the stability required for longer 3D-experiments.

**Fig 1:**
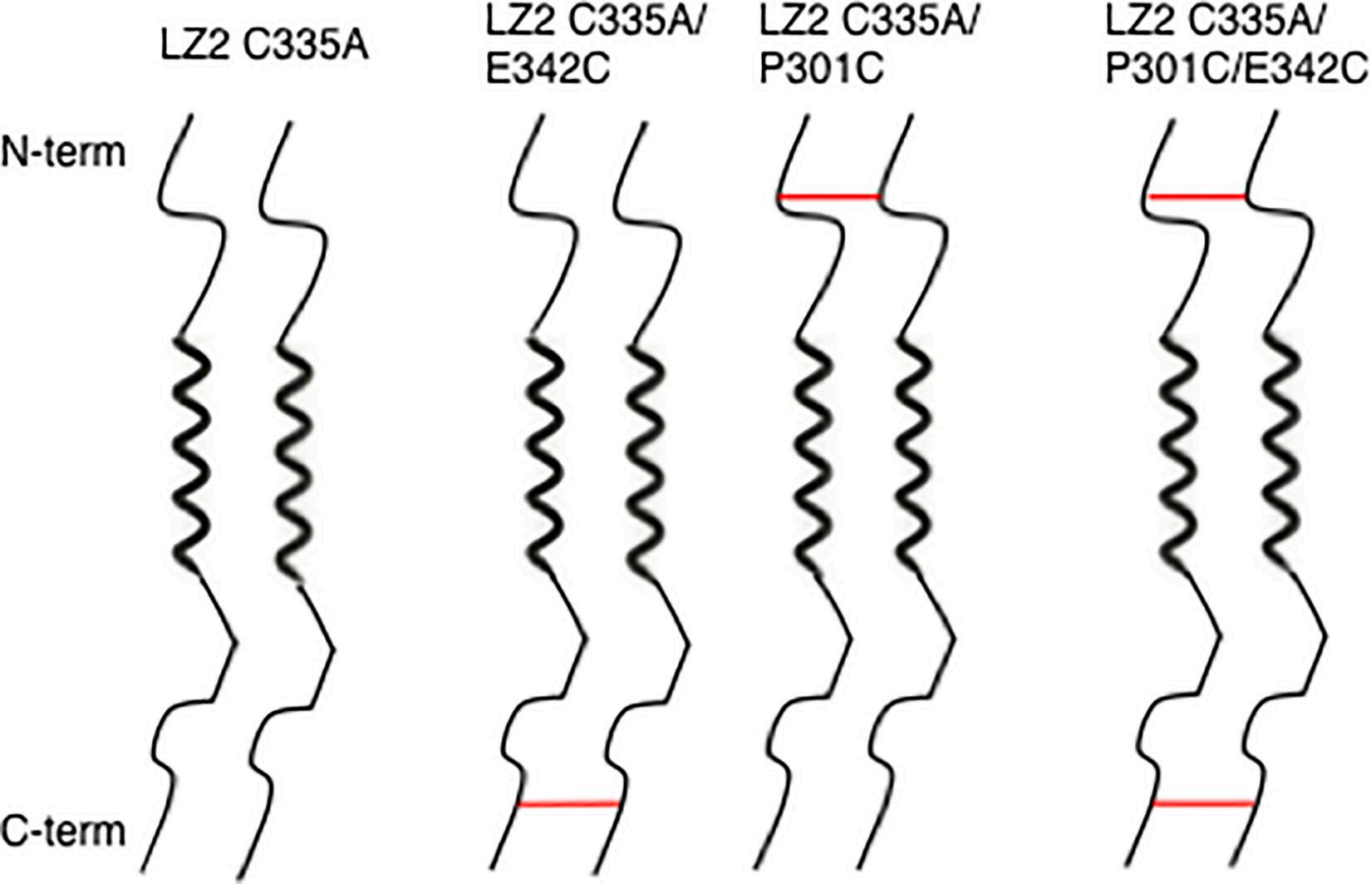
Schematic illustration of different cysteine mutants of the Nek2 leucine zipper with disulfide bonds (shown as red lines) placed at different positions near the N- or C-terminus. Thick zig-zag lines indicate the folded, dimeric coiled-coil helical core while the thin lines top and bottom indicate unfolded, flexible portions of the protein.

In order to address these limitations, a different approach was taken in this study. Instead of introducing disulfide bonds into the folded core of the protein to “lock” one of the two conformations, disulfide bonds were introduced into the flexible regions flanking the leucine zipper (see Fig 1). The positions for these were chosen based on the experimental results for the LZ5 C335A/K309C mutant from our earlier work in combination with results from sequence analysis. It was envisaged that such a disulfide bond would reduce the mobility of the terminal regions and thus introduce additional friction into the process of conformational exchange. Consequently, this intervention was expected to affect only the dynamics of the conformational exchange without affecting the equilibrium populations. We therefore termed these disulfide bonds ‘kinetic’ to distinguish them from the previous ‘thermodynamic’ ones. The expected advantages of this ‘kinetic’ approach were a significant reduction in the exchange rate and thus more amenable linewidths with spectra that are virtually identical to those of the wildtype. In addition, this approach should allow the simultaneous study of both conformers because the position of the equilibrium is not affected.

Importantly, this different use of a disulfide bond produced marked effects on the conformational exchange dynamics and as a result of that on the NMR spectra recorded with the Nek2 leucine zipper. Thus, severe line broadening and the loss of signal occurring in 2D and 3D NMR spectra due to exchange-related shortening of transversal relaxation time (T2) was substantially attenuated (see Fig 1(A) and 1(B)). Also, coherence transfer leakage, which happens as a result of conformational exchange in the course of a long NMR experiment (e.g. HNCACB), was eliminated. Overall, the exchange regime was pushed from a slow/intermediary timescale, which is particularly unfavorable for NMR, towards a much slower timescale. This study represents the first application of this important tool for the targeted modulation of the conformational exchange kinetics of a biological model system.

## Methods

### Construct generation

All protein expression constructs were cloned into pLEICS07 vectors. Inserts were generated by means of polymerase chain reaction by using 2.5 units of Platinum Pfx DNA Polymerase (Roche) with 200 ng of template, 500 nM of each primer, 1.2mM dNTP mix, and 1mM MgSO4 on a Techne TC-312 thermocycler. Amplification was achieved by initial denaturation for 2 minutes at 94°C followed by 30 cycles of 15 seconds melting at 94°C, 60 seconds at 60°C and 30 seconds extension at 68°C.

PCR products were purified with QIAquick PCR purification kit according to the manufacturer’s protocol. Purified PCR products and pETM-11 target vector DNA were double digested with NcoI and BamHI. Purification of the product of vector digestion occurred by electrophoresis (1% agarose gel, Melford Labs). A QIAquick gel extraction kit was used to extract DNA from excised bands. 50 ng of gel purified digestion product from the vector and 150 ng of digested PCR product were mixed and ligated using the rapid DNA ligation kit (Roche). 1/10 of the ligation reaction was transformed into 100μl DH5a-T1R chemical competent cells (Invitrogen). The coding sequence of Nek2 from residue 299Ser to 343Asp with two additional N-terminal residues (297Gly and 298Ala) was cloned into a pLEICS07 vector carrying a kanamycine resistance.

Protein sequence of different mutants (cloning artefacts are indicated by italic formatting, added/removed cysteins are underlined):

LZ2 C335A: *GA*SSPVLSELKLKEIQLQERERALKAREERLEQKEQEL**C**VRERLAED

LZ2 C335A/E342C: *GA*SSPVLSELKLKEIQLQERERALKAREERLEQKEQEL**A**VRERLA**C**D

LZ2 C335A/P301C: *GA*SS**C**VLSELKLKEIQLQERERALKAREERLEQKEQEL**A**VRERLAED

LZ2 C335A/P301C/E342C: *GA*SS**C**VLSELKLKEIQLQERERALKAREERLEQKEQEL**A**VRERLA**C**D

Different mutants of the initial insert LZ2 C335A were generated by means of site-directed mutagenesis. Point mutations were generated using the QuikChange kit (Stratagene) using the manufacturer’s protocol. For all constructs and mutants, small scale cultures were grown for several positive clones, and DNA was prepared with the Qiagen miniprep kit. Accuracy of vector and insert was checked by DNA sequencing.

### Protein preparation and NMR samples

BL21* cells were transformed with DNA of the respective construct. The cells were then grown in LB at 37°C up to an OD600 of 0.8, when LB was exchanged by M9 minimal medium. 15N-single labelled protein, samples were produced by adding 15N-NH4Cl as sole source of nitrogen to the M9 minimal medium, while 15N-NH4Cl and 13C-glucose were added to produce 15N/13C-double-labelled samples. Cells were lysed by French press in a lysis buffer containing 20 mM Na2HPO4/NaH2PO4-buffer, 500mM NaCl, 10mM imidazole, 0.02% NaN3 and 1 aliquot of DNAse (1mg/ml). For the purification of all proteins a two-step procedure comprising a His Trap nickel-charged IMAC column and a size exclusion column was used. This was either achieved by a gravity column filled with Ni Sepharose 6 Fast Flow resin followed by AKTA purification with a Sephadex 16/70 gel filtration column (GE healthcare) or as an integrated in-house automatic protein purification system (Gilson HPLC, devised by Dr A Alexandrovich).

After dialysis with NMR buffer (20 mM sodium phosphate, 50mM NaCl, 0.02% NaN3, pH 7.0), samples were concentrated with Vivaspin 20 concentrators (Sartorius) with a molecular weight cutoff (MWCO) of 3 kD. In the appropriate samples disulphide bond formation was encouraged during dialysis (in the same buffer but with an elevated pH of 8.0) using spectrapore tubing with a MWCO of 1 kD by bubbling air through the buffer for 1h at room temperature. Formation of disulphide bonds was probed by reducing/non-reducing SDS-PAGE (NuPage). Final NMR samples contained 280 μl of protein and 30 μl D2O in a Shigemi tube. Concentrations of protein samples used for backbone experiments ranged between 0.5 and 1.0 mM. Concentrations of samples used to study conformational excahnge were between 1.0 and 2.0 mM. Because constructs lacked aromatic residues, concentrations were determined by means of Qubit protein concentration assay (Invitrogen).

### NMR spectroscopy, spectra processing and assignments

NMR-spectra were recorded on Bruker-Avance spectrometers with the following magnetic field strengths: 500 MHz, 700 MHz, 800 MHz and 900 MHz, fitted with helium cooled cryoprobes. For most NMR experiments, standard Bruker sequences were used. Exchange experiments were performed with a version of the original experiment to monitor slow exchange [8], modified as described previously [6] and similar to other methods directed at improving exchange experiments [9]. In the modified version of this experiment, initial excitation of the proton is followed by indirect frequency labelling. To achieve this, in the pulse sequence shown in Fig 1 of [8] an incremented t_1_ delay was added to the initial τ_a_ periods while the t_1_ periods on nitrogen were replaced by τ_b_. Subsequently, magnetization is transferred to the nitrogen, where magnetization is rotated onto the z-axis. Nitrogen Z-magnetization (N_z_) is then allowed to exchange during a mixing time T. Then, N_z_ is rotated onto the xy-plane and transferred back to the proton where it is detected. These experiments are recorded as simple NOESY-like 2D spectra with two proton axes or as pseudo-3D spectra where the third axis is the mixing time.

NMR data was processed by means of Topspin 3.1 software. Visualization, analysis and assignment of all spectra was done with CCPNmr analysis 2.4 [10]. Peak integrals and linewidths were extracted automatically in CCPNmr using a parabolic fit to determine peak positions and a truncated box sum to calculate to integral. Exchange kinetics were analysed using home written Mathematica and R scripts as described previously [6] based on the original equations proposed by Kay and coworkers (equations 1a/1b for the time dependency of the auto-peak intensities and equations 1c/1d for the cross-peak intensities taken from [8]). The Arrhenius analysis was performed with GraphPad Prism 8.0 using the logarithmic version (Eq 2) of the standard equation for the temperature dependence of a rate constant (Eq 1) [11]:

$$k_{ex}=k_{ex}^{\prime }*e^{\Delta G^{‡}/RT}$$ 1

$$ln\;k_{ex}=ln\;k_{ex}^{\prime }+\frac{\Delta G^{‡}}{RT}$$ 2

Where k_ex_ is the exchange rate constant, k’_ex_ the pre-exponential factor of the rate equation also known as the maximal exchange rate constant, ΔG^‡^ is the activation energy, R the universal gas constant and T the absolute temperature. The equation is being linearized using its logarithmic version so that a plot of ln k_ex_ against 1/T should give a straight line. From the slope of this line the activation energy and from the y-intercept the pre-exponential factor are extracted.

### CD spectroscopy

UV and CD spectra were acquired on the Applied Photophysics Chirascan Plus spectrometer (Leatherhead, UK). A 0.5mm Quartz Suprasil rectangular cell (Hellma UK Ltd) was employed in the region of 260–180 nm. The instrument was flushed continuously with pure evaporated nitrogen throughout the experiment. The following parameters were employed: 2 nm spectral bandwidth, 1nm step size and 1.0 s accumulation time per point. The far-UV CD spectrum was buffer baseline corrected and measured at 23°C. Secondary structure content was analysed by a home written Mathematica (Wolfram) script by least squares fitting the experimental spectra to linear combinations of standard spectra of α-helix, β-sheet and random coil. For denaturant chemical stability analysis 10 M urea and 8 M guanidinium hydrochloride (GdnHCl) stock solutions were prepared. Titrations were performed by incrementally adding the appropriate volumes of urea respectively GdnHCl and by recording molar ellipticities at 222 nm along specific titration points.

## Results

### Generation of different mutants of the Nek2 leucine zipper with different positions of disulfide bonds

**Fig 2:**
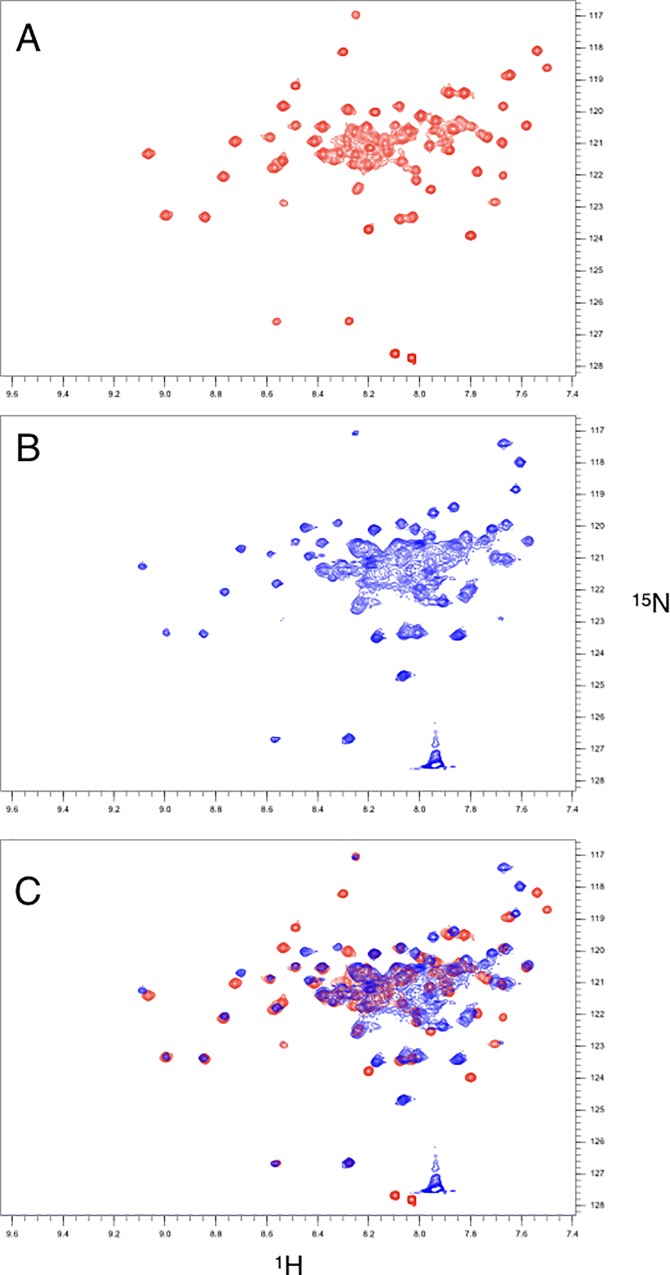
^15^N-HSQC spectra of Nek2 leucine zipper mutants. **A)** LZ2 C335A **B)** LZ2 C335A/E342C. A magnified view of selected peaks to illustrate exchange-related line broadening of the same spectral region is shown in both spectra. **C)** Superimposition of spectra shown in (A) and (B).

We used site-directed mutagenesis to produce a total of 5 different cysteine mutants of the LZ2 construct of the Nek2 leucine zipper. As in our previous work of introducing disulfide bonds into the Nek2 leucine zipper (REF) we first removed the native cysteine in position 335 (C335A) to remove any potential interference. The mutant LZ2 C335A is therefore representative of the wild type. Of these constructs, however, only LZ2 C335A/P301C, LZ2 C335A/E342C and LZ2 C335A/P301C/E342C expressed at levels sufficient to produce samples for NMR studies. Expression levels of LZ2 C335A/S300C and LZ2 C335A/D343C were too low (data not shown). Fig 1 shows a schematic representation of the wild type Nek2 leucine zipper and the 3 mutants that could be investigated further. A first view of the effect of the introduction of a disulfide bond is shown in Fig 2.

### Probing the formation of disulfide bonds

The existence of disulfide bonds was probed by SDS-PAGE, the analysis of cysteine residue ^13^C_β_ chemical shifts and CD spectroscopy (see S1 Fig). Samples of LZ2 C335A/E342C and LZ2 C335A/P301C were subjected to non-reducing SDS-PAGE as they were after purification or after additional incubation and boiling in 10 mM β-mercaptoethanol. Both mutants in the oxidized version appeared predominantly (estimated > 80%) at an apparent molecular weight of around 12 kD with only very little appearing around 6 kD (estimated < 20%). In contrast, for the reduced samples the higher molecular weight band was much weaker (and the lower molecular weight band was much increased (estimated around 50% each) with a substantial amount of smeared protein in between the two bands. With monomer/dimer molecular weights of around 5.5 and 11 kD for the LZ2 construct [6] the observed bands represent quite well the monomeric and dimeric states of the protein. The predominant appearance of the higher molecular weight band in the oxidized samples confirms the formation of disulfide bonds while the weaker intensity of the higher molecular weight band in the reduced samples suggests that the disulfide bonds have been broken. The fact that we still see some of the protein in the higher molecular weight band could be due to incomplete reduction of the disulfide bonds or residual stability of the noncovalent dimeric assembly of the leucine zipper even under strongly denaturing conditions. It is well known that some coiled-coils can resist the usual means of denaturation quite well [12] and we regularly observe dimer bands for Nek2 LZ constructs, even those without disulfide bonds (R. Croasdale, D. Gutmans, M. Pfuhl, unpublished).

Chemical shifts of cysteine C_β_ resonances are a good indicator of whether the respective cysteine occurs in reduced form or makes part of a disulfide bond [13]. The chemical shifts of both C_β_-signals in a HNCACB experiment are above 40 ppm which clearly suggests the formation of a disulfide bond by the respective cysteine residues. For reduced cysteines C_β_ chemical shift values around 30 ppm would be expected (S1B Fig).

The effect of the disulfide bonds on the thermal stability of the coiled coils was measured by CD spectroscopy unfolding curves (S1C Fig). The control construct LZ2 C335A has virtually identical melting temperatures of 42.4°C and 41.7°C in the absence and presence of DTT, respectively. In marked contrast, the LZ2 constructs with engineered disulfide bonds had significantly increased melting temperatures in the absence of DTT (C335A/P301C: 71.2°C, C335A/E342C: 75.5°C, C335A/P301C/E342C: 70.3°C) while they came back to the level of wt in the presence of DTT (C335A/P301C: 42.9°C, C335A/E342C: 44.3 ^o^C, C335A/P301C/E342C: 41.4°C). We assume that the increase in stability is the result of the formation of the disulfide bond(s) and thus indicates that the disulfide bonds have indeed formed.

To ensure that the formation of the disulfide bonds only affects the exchange kinetics and not the equilibrium of the conformers we measured the peak volumes of exchanging pairs of peaks corresponding to individual amino acids in both conformers (S1D Fig). There is no difference in the ratio–very close to 1 –between LZ2 C335A and LZ2 C335A/E342C. We also probed the effect of the absence and presence of the disulfide bond in LZ2 C335A/E342C and noticed (as shown in S1E Fig) that peak positions, especially for residues in the helical core of the protein, are barely affected by presence or absence of the disulfide bond. In contrast, the linewidths are considerably different.

### Probing conformation and stability

**Fig 3:**
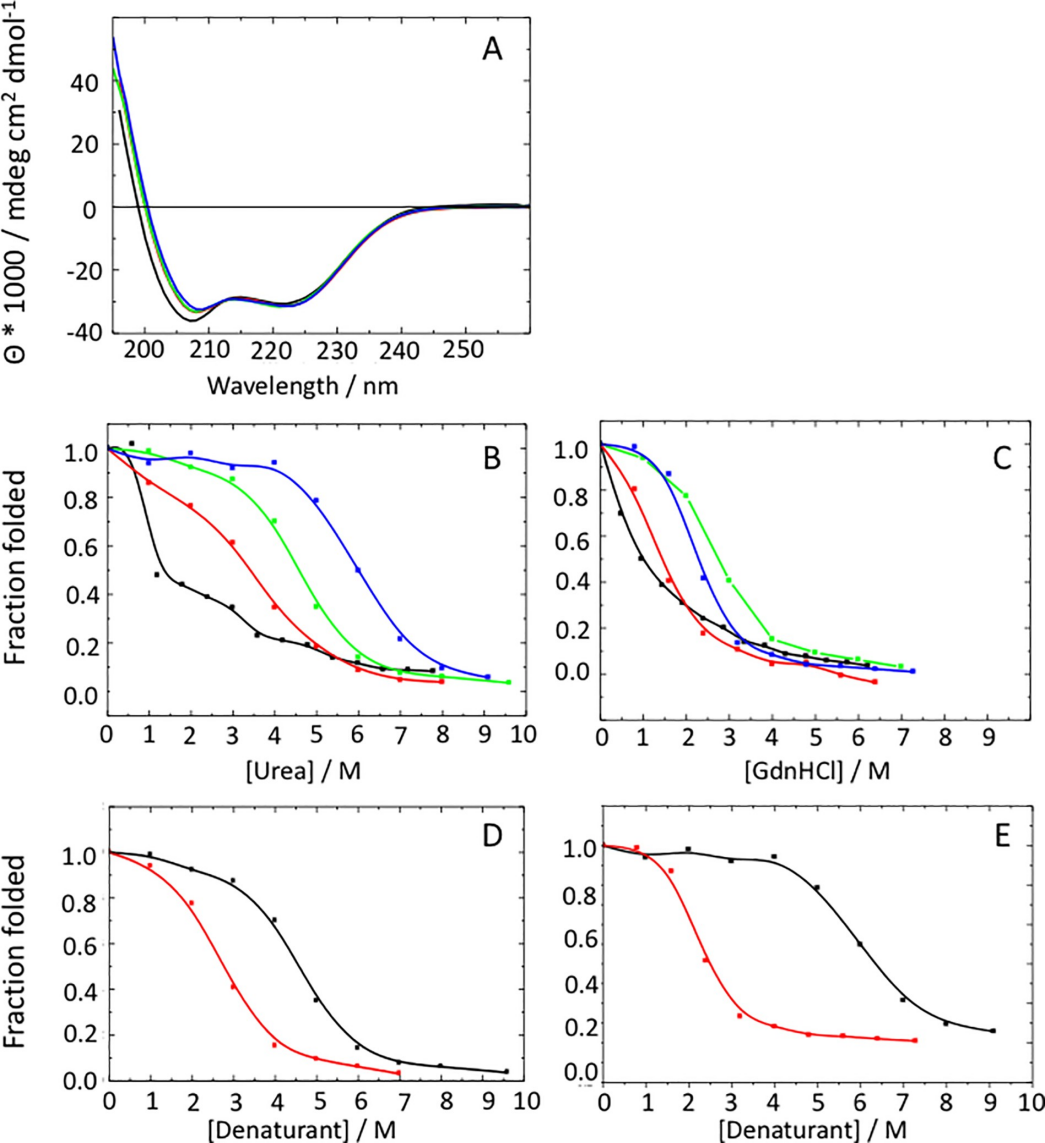
**A)** Superimposition of CD spectra recorded for LZ2 C335A (black), LZ2 C335A/P301C (red), LZ2 C335A/E342C (green) and LZ2 C335A/P301C/E342C (blue). **B)** Superimposition of plots of denaturant titration carried out with 10 M urea for LZ2 C335A (black), LZ2 C335A/P301C (red), LZ2 C335A/E342C (green) and LZ2 C335A/P301C/E342C (blue). **C)** Superimposition of plots of denaturant titration carried out with 8M GdnHCl for LZ2 C335A (black), LZ2 C335A/P301C (red), LZ2 C335A/E342C (green) and LZ2 C335A/P301C/E342 (blue). **D)** Comparison of Urea (black) and GdnHCl (red) denaturation curves of LZ2 C335A/E342C. **E)** Comparison of Urea (black) and GdnHCl (red) denaturation curves of LZ2 C335A/P301C/E342C.

For all mutants CD spectra were recorded at room temperature in order to assess their α-helical content. Fig 3A and 3B show superimpositions of the respective plots of LZ2 C335A, LZ2 C335A/E342C, LZ2 C335A/P301C and LZ2 C335A/P301C/E342C. The graphs of all mutants display highly negative minima ellipticity at 208 nm and 222 nm and highly positive values at 195 nm in good agreement with a high α-helical content. Secondary structure analysis gave helical contents of 61 ± 4% for LZ C335A, 63 ± 4% for LZ2 C335A/E342C, 62 ± 4% for LZ2 C335A/P301C and 64 ± 4% for LZ2 C335A/P301C/E342C in good agreement to the expected coiled-coil structure of the construct. Very little variations were seen in the spectra or the derived alpha α-helical contents.

In addition to the thermal stability tests used to probe the formation of the disulfide bonds we also performed chemical denaturation stability studies in order to assess the effect of the disulfide bonds on the stability of each mutant in more detail.

The chemical stability of the different mutants was probed with both 8 M GdnHCl and 10 M Urea. In these studies, molar ellipticities at 222 nm were measured as a function of the concentration of the respective denaturant. As shown in Fig 3C and 3D and Table 1, the mutants differ considerably with respect to urea concentration that is required to unfold half of the protein. Unfortunately, due to technical reasons, no [Urea]_1/2_ and [GdnHCl]_1/2_ midpoints could be determined for LZ2 C335A.

**Table 1:** [Urea]_1/2_ and [GdnHCl]_1/2_ midpoints of different LZ2 mutants as determined by CD spectroscopy.

LZ2 mutant	[GdnHCl]_1/2_ [M]	[Urea]_1/2_ [M]
LZ2 C335A	-	-
LZ2 C335A/P301C	1.24	3.27
LZ2 C335A/E342C	2.68	4.52
LZ2 C335A/P301C/E342C	2.24	5.97

The following order of the different mutants results from an analysis of chemical stability carried out with 10M urea: LZ2 C335A/P301C/E342C > LZ2 C335A/E342C > LZ2 C335A/P301C > LZ2 C335A (see Table 1). This order suggests that the introduction of a disulfide bond increases the chemical stability and the achieved gain of stability is additive for multiple disulfide bonds. Another denaturant titration study conducted with 8M GdnHCl provides a slightly different picture. While LZ2 C335A/P301C and LZ2 C335A remain the least sTABLE, the order between LZ2 C335A/E342C and LZ2 C335A/P301C/E342C is reversed. (see Table 1). [GdnHCl]_1/2_ and [urea]_1/2_ midpoints of LZ2 C335A/P301C/E342C are almost twice as large than those of LZ2 C335A/P301C. The [Urea]_1/2_ midpoints of LZ2 C335A/P301C/E342C is also considerably larger than that of LZ2 C335A/E342C. However, the [GdnHCl]_1/2_ midpoint of C335A/P301C/E342C is smaller than that of LZ2 C335A/E342C.

It has previously been reported that the ionic nature of GdnHCl with its potential to mask interhelical electrostatic interactions can result in substantial differences between denaturation curves obtained with GdnHCl and those obtained with urea [14]. As a consequence, chemical stabilities determined with urea provide a more comprehensive picture of the forces that need to be disrupted in order to unfold a coiled coil. In particular, the gap between the [GdnHCl]_1/2_ and [Urea]_1/2_ midpoints make it possible to assess the contribution of interhelical electrostatic interactions to the stability of the three-dimensional protein structure. Comparing the different denaturation curves makes it clear that the introduction of a second disulfide bond widens the gap between the stability of the different mutants. This suggests that the relative contribution of interhelical electrostatic interactions to all stabilizing forces is considerably larger in LZ2 C335A/P301C/E342C than in LZ2 C335A/E342C.

### Assignment of NMR spectra

**Fig 4:**
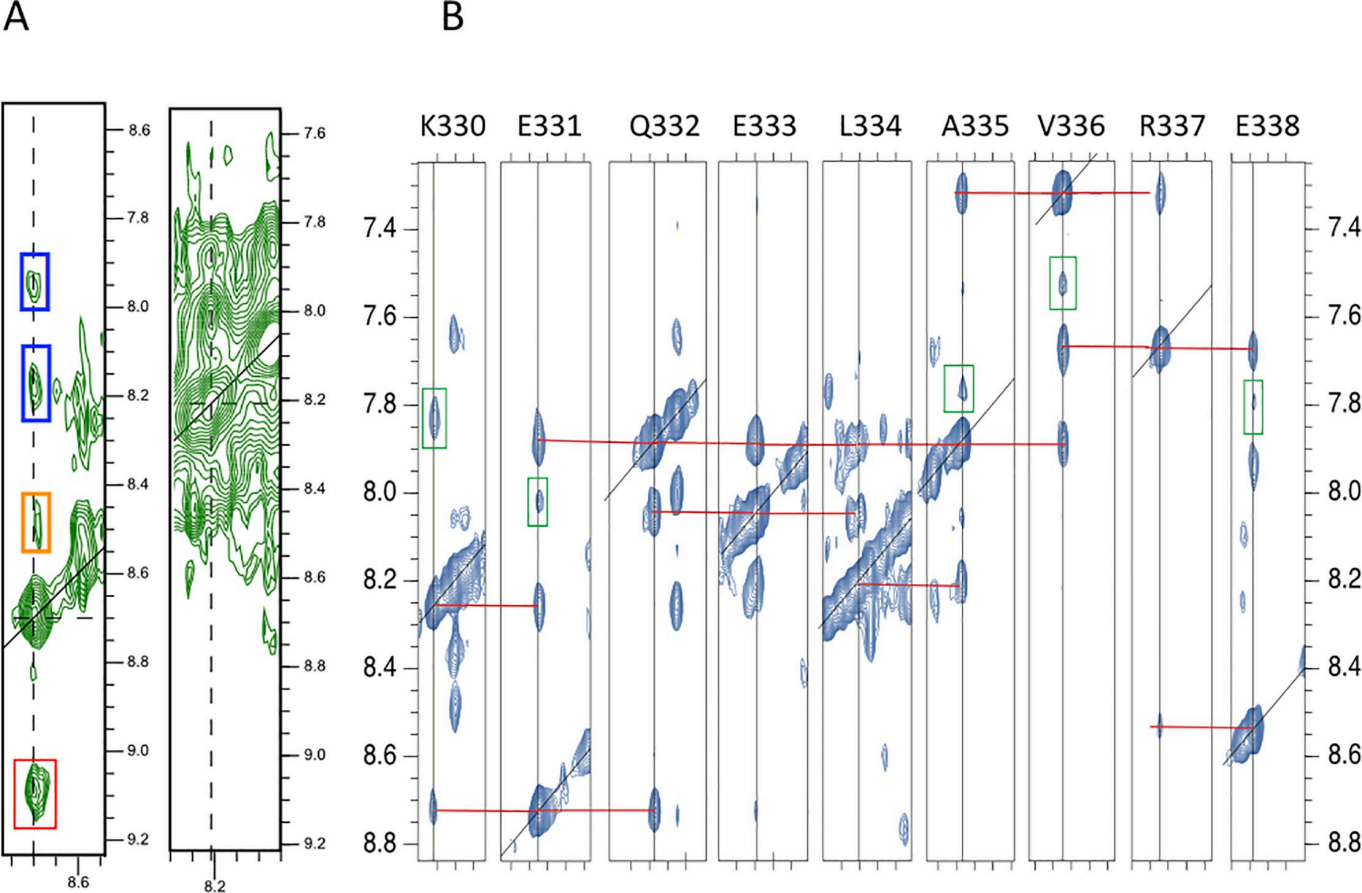
**A)** ^15^N-NOESY-HSQC-strips of LZ2 C335A showing genuine NOE cross peaks (blue boxes), exchange cross peaks (red boxes) and coherence transfer leakage (orange boxes), overcrowding and severe line broadening in the amide-amide-region of the 3D ^15^N-NOESY-HSQC experiment. **B)** ^15^N-NOESY-HSQC-strips of residues K330 to E338 in the (B) conformation of LZ2 C335A/E342C aligned in sequential order. Note that throughout this work we shall be labelling the two conformers with (A) or (B). In each strip, connectivities between sequential amide-auto- and cross peaks are highlighted in red, while cross-peaks stemming from exchange are indicated in green boxes. Note that in B) the genuine NOESY cross peaks are more intense than the exchange cross peaks which is the other way around in A).

Short distances between sequential amides (H_N_,H_N_(i,i+1)) resulting in strong amide-amide NOEs are among the hallmarks of NMR spectra of α-helices [15,16]. The ^15^N-NOESY-HSQC-strips of the WT-analogue LZ2 C335A presented in Fig 4A illustrate some of the difficulties encountered with establishing amide-amide-connectivities for this mutant. Coherence transfer leakage makes cross-peaks related to the partner of a pair of exchanging residues appear, thereby adding further complications to the assignment. Overcrowding combined with severe line broadening makes an unambiguous identification of amide-amide-connectivities impossible.

**Fig 5:**
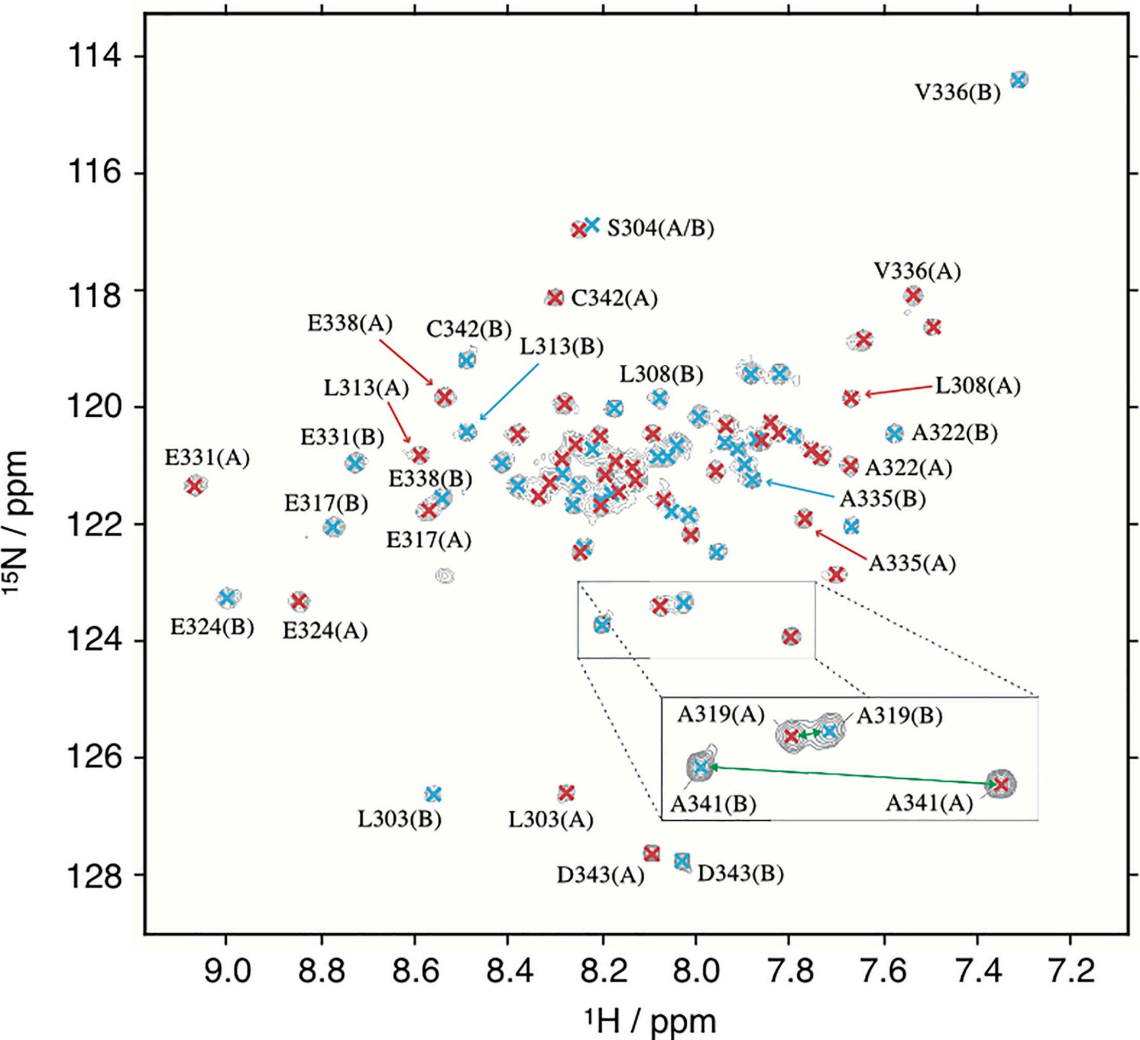
A ^15^N−HSQC of LZ2 C335A/E342C. Signals corresponding to conformation (A) are coloured red, and signals corresponding to conformation (B) are coloured blue.

Fig 5B shows the ^15^N-NOESY-HSQC-strips of residues K330 to E338 in the (B) conformer of LZ2 C335A/E342C arranged in sequential order. In each strip, connectivities between sequential amide-auto- and cross-peaks are highlighted in red, while cross-peaks stemming from exchange are indicated in green boxes. Interestingly, sequential amide-amide cross-peaks display greater intensities than their exchange-related counterparts. The lack of amide-amide NOEs between more distant residues along the same α-helix (H_N_,H_N_(i,i+2)) can be explained by the conformational exchange dynamics. The sequential order of all residues for both conformers could be established using amide-amide-connectivities as seen in the ^15^N-NOESY-HSQC. Subsequently, HNCACB/HNcoCACB, HNCA/HNcoCA and HNCB/HNcoCB (essentially a HNCACB where the entire magnetization is relayed on the C_β_) not only provided an alternative strategy to verify sequential assignments but also yielded chemical shifts for the majority of α-carbon (C_α_ & C_β_) resonances. Thus, the entire backbone and numerous H_β_/C_β_ resonances could be assigned (See S1 Table) with only a few exceptions. Assignment difficulties and ambiguities mainly arose because of two factors: firstly, a scarcity of signals in numerous HNCACB-strips where only intraresidual signals (i) could be observed while interresidual signals (i,i-1) were missing can be attributed to the conformational exchange dynamics. Secondly, poor chemical shift dispersion, a characteristic of α-helices, leads to a crowded central region of the ^15^N-HSQC, which resulted in significant signal overlap in some 3D experiments.

For residues S299 –D343 of both conformations, the complete backbone assignment has been achieved, including C_β_ and H_β_ resonances with only very few exceptions. An indication of the distribution of assignments of both conformers in the HSQC experiment is shown in Fig 5. In total the following nuclei were assigned: H_N_, N_H_, C_α_, H_α_, C’ C_β_, H_β_. An overview of assigned spins is given in the S1A and S1B Tables of the Supporting Material.

### Secondary structure analysis based on chemical shifts

**Fig 6:**
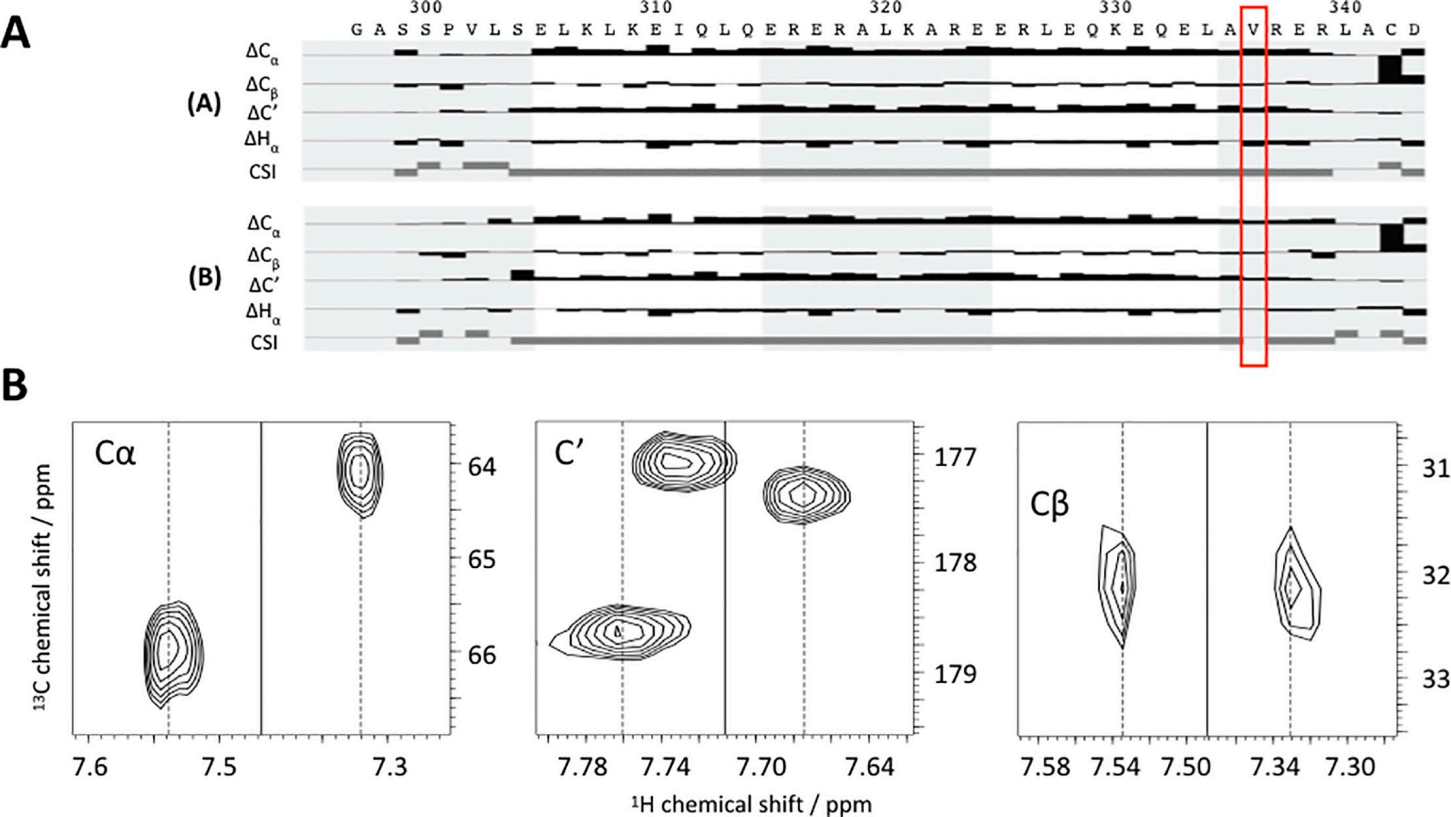
**A)** Secondary chemical shift analysis of both conformers of LZ2 C335A/E342C. The CSI values are calculated for a combination of all four of the shown secondary chemical shifts. The significant change in the secondary structure at V336 (CSI going from -1 (= helical) in conformer (A) to 0 (= undefined) in conformer (B)) is indicated by a red box. Illustration of chemical shift differences of V336 C_α_, C_β_ and C’ resonances in both conformers taken from strips in the 3D HNCACB and 3D HNCO experiments, respectively. **B)** C_α_ has a chemical shift difference larger than 1.5 ppm. **C)** C_β_ chemical shifts are very close. **D)** Strips taken from the HNCO experiment show a chemical shift difference for the C’ resonance of around 1 ppm between the two conformers. For all nuclei conformer (A) is on the left, conformer (B) is on the right.

Chemical shifts have been shown to provide vital information on wider range and factors related to the local magnetic environment of specific spins. Especially secondary chemical shifts, the difference of an observed resonance from the chemical shift it is expected to have in a random coil, have been established as reliable indicator for secondary structures, such as α-helices and β-sheets [17,18]. Based on the complete backbone assignment, the secondary structure of the two conformers of LZ2 C335A/E342C could be determined by means of secondary shifts. Secondary chemical shifts were calculated in CCPNmr for the following nuclei: H_N_, N_H_, C_α_, C_β_, C’, H_α_ for all residues with a few exceptions (for G297 and A298 no spins could be assigned at all and as expected for proline residues no amide-group spins could be assigned for P301) [10,19]. Fig 6A shows a secondary structure chart produced by CCPNmr for both conformers [17]. The charts clearly show α-helical regions extending from residue S304 to R339 for both. Intriguingly, in conformation (B), the helix is interrupted at V336 while it continues all the way to the end in the (A) conformer. Fig 5 illustrates the distance between the peak for this signal and its exchange partner in a ^15^N-HSQC. Because V336(B) is the only residue that interrupts the α-helical stretch in either conformation and because of its exceptional position in the ^15^N-HSQC, a comparison of the backbone carbon resonances of V336 is shown in Fig 6B. It should be noted that V336 is not the only residue with significant chemical shift differences amongst the amide resonances of both conformers. Already a superficial inspection of Fig 5 reveals that e.g. L303, E331 and A341 also experience a large difference in amide proton chemical shift. However, none of these show similar differences in the secondary structure specific chemical shifts.

### Differences of chemical shifts between corresponding spins of each conformation

**Fig 7:**
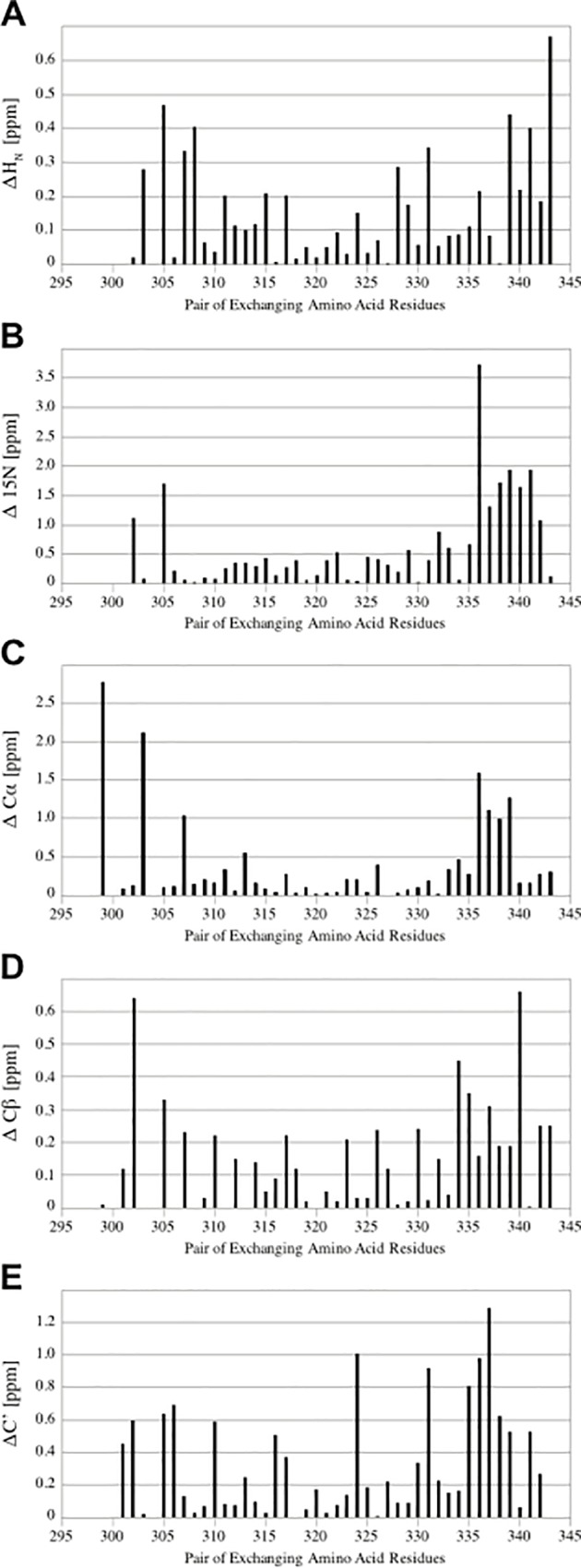
**Chemical shift differences of exchanging pairs of resonances between the two conformations of LZ2 C335A/E342C for a range of different resonances: A)** Η_Ν_. **B)** Ν_Η_. **C)** Cα. **D)** Cβ**. E)** C’.

A comprehensive analysis of the chemical shifts of the backbone spins including does not only allow an analysis of secondary structure but also opens a window into the subtle structural differences between the two conformations, as demonstrated for V336 in Fig 6. For that purpose, a detailed overview of the chemical shift differences between exchanging pairs of residues was generated for H_N_, N_H_, C_α_, C_β_ and C’ resonances (Fig 7). Fig 7A and 7B show somewhat unexpectedly that amide groups of residues belonging to the flexible C-terminal region experience larger ^1^H and ^15^N-chemical shift changes than those of other residues. As previously pointed out, V336 represents an exception with respect to the substantial difference of all assigned resonances.

As indicated in Fig 7B and 7C, the N_H_ and C_α_ chemical shift changes experienced by residues belonging to the core region of the coiled coil (residues S304 –A335) show a relatively flat distribution with a few exceptions (ΔC_α_ K307). In contrast, residues positioned at the flexible N-terminus show a rather heterogeneous picture. For L303, a marked difference in their C_α_ is accompanied by a relatively large gap in their amide proton chemical shifts. For other residues, such as S299, a large difference in their C_α_-chemical shift is not emulated by any others of its resonances. In Fig 7E, a group of residues with larger chemical shift differences of their carbonyls can be detected (A335-R339). This is likely related to the fact that the residues flanking the C-terminus of the coiled coil (L340 –D343) do not adopt α-helical configuration and are therefore not available as partners for the formation of characteristic hydrogen bonds [20,21]. As a consequence, alternative bonding partners have to be found in order to satisfy the carbonyl groups of these residues. C-cap motifs can occur in very different structural arrangements, and the geometry of hydrogen bonds impacts on the ^13^C chemical shifts of involved carbonyl spins [22–24]. Therefore, it is likely that the large ^13^C chemical shift changes among carbonyl spins reflect different C-capping motifs occurring in the two conformers of LZ2 C335A/E342C.

### Analysis of linewidths

**Fig 8:**
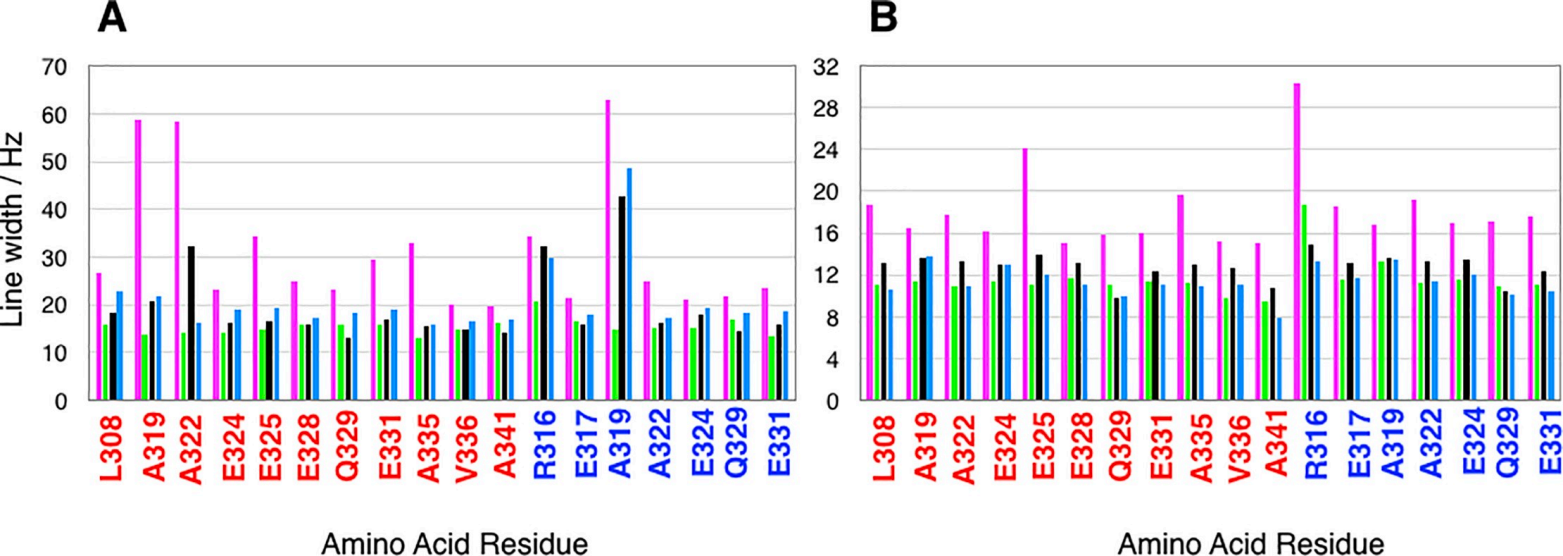
Analysis of linewidths of selected signals in ^15^N-HSQC spectra. All experiments were recorded at 700 MHz and 298K. Bars for LZ2 C335A are coloured magenta; LZ2 C335A/E342C green; LZ2 C335A/P301C black; LZ2 C335A/P301C/E342C blue. **A)** H_N_ linewidths **B)** N_H_ linewidths. Residue labels are coloured by conformer: (A) is red and (B) is blue.

One of the most obvious signs of conformational exchange in 2D and 3D NMR spectra is line broadening. In order to analyse, how the different timescales on which conformational exchange occurs in all 4 mutants translates into line broadening, a ^15^N-HSQC spectrum was recorded at 700 MHz and 298K for each of them. A number of signals that are equally well-resolved in all 4 spectra was selected and ^1^H and ^15^N-linewidths were measured. This data, which is presented in Fig 8, clearly shows that linewidths measured for LZ2 C335A in both ^1^H and ^15^N-dimension significantly exceed those determined for the other 3 mutants. A closer inspection of these graphs reveals two more subtle trends. With respect to the ^1^H dimension, linewidths obtained for LZ2 C335A/P301C/E342C are often slightly larger than those of LZ2 C335A/E342C and LZ2 C335A/P301C. However, LZ2 C335A/P301C frequently shows slightly elevated linewidths in ^15^N-dimension compared to LZ2 C335A/E342C and LZ2 C335A/P301C/E342C. Based on the measured linewidths and the overall appearance of the ^15^N-spectra, LZ2 C335A/E342C was selected for all subsequent NMR analyses, including backbone assignment.

### Analysis of conformational exchange

**Fig 9:**
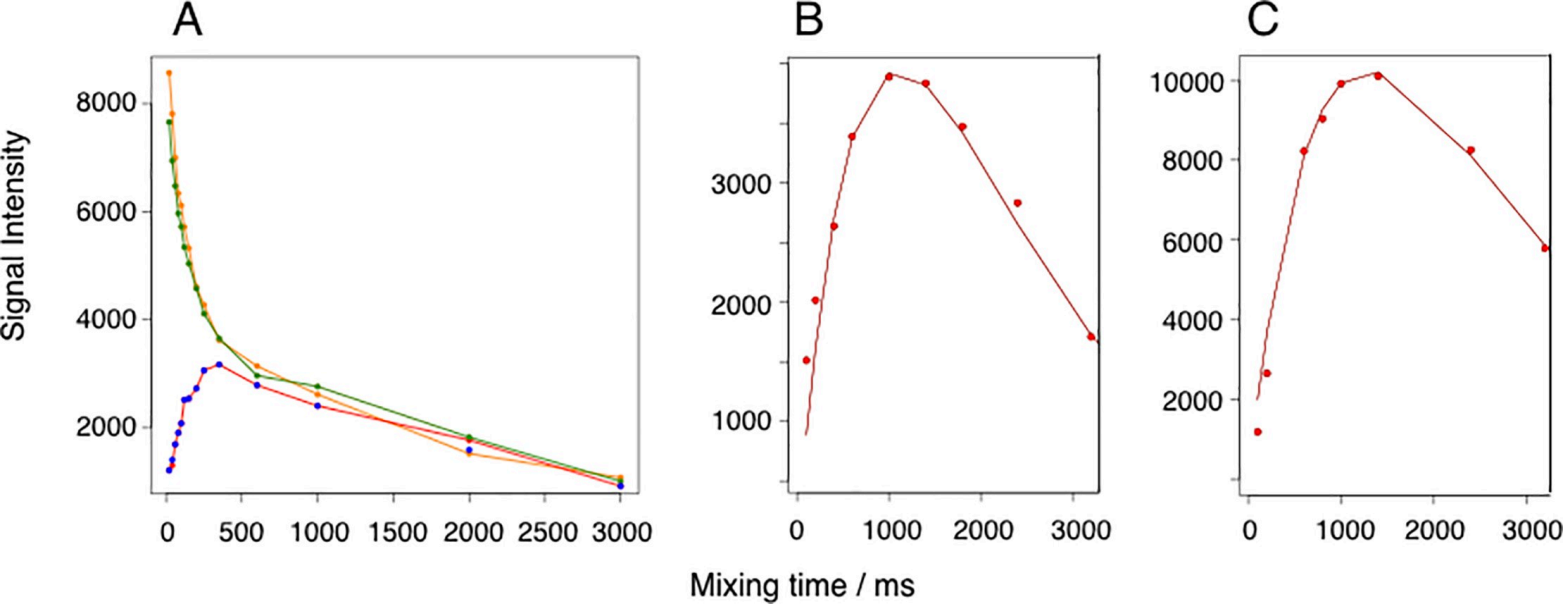
**A)** Representative plot for an exchanging pair of residues with 2 auto peak-curves (in orange and green) and 2 cross-peak curves (in blue and in red). Note that the latter are right on top of each other. This data was extracted for E331 in LZ2 C335A/E342C. **B)** Graph of a fitted plot of cross-peak intensity as a function of mixing time for E324 of LZ2 C335A/E342C. Extracted exchange frequency: 7.0 s^-1^. **(C)** Graph of a fitted plot of cross-peak intensity as a function of mixing time for R339 of LZ2 C335A/P301C/E342C. Extracted exchange frequency: 5.7 s^-1^. All data obtained at 298K.

Chemical exchange occurs when a nucleus samples different magnetic environments as a result of conformational changes or chemical reactions. Conformational dynamics occur in a large number of proteins and play a crucial role for protein function [25]. NMR is ideally suited to investigate protein dynamics. Chemical exchange affects a range of NMR parameters, such as chemical shift, line width, transversal relaxation and signal intensity. Importantly the way these parameters are affected by exchange considerably depends on its timescale. The NMR timescale is defined as the relation between the frequency difference a spin samples in different environments and the exchange rate. In this study, exchange rates were determined for LZ2 C335A, LZ2 C335A/E342C and LZ2 C335A/P301C/E342C by using a heteronuclear correlation experiment previously described [6]. A representative plot for the time dependency of the exchange and auto peaks in this experiment for an exchanging pair of residues is shown in Fig 9A. As described earlier, exchange rates were extracted by fitting the experimentally obtained data to the mathematical model presented previously [8]. A fitted plot for the pair of exchanging residues of E324 of LZ2 C335A/E342C is shown in Fig 9B while Fig 9C displays a fitted plot for R339 of LZ2 C335A/P301C/E342C. A similar approach was also attempted for LZ2 C335A. However, as shown for the representative example of A341 of LZ2 C335A in Fig 9A the ascent of the signal intensity can be well fitted with the underlying mathematical model, but the signal decay cannot (see also S2 Fig). The course of the signal decay suggests that it is governed by at least two factors. This leaves the possibility that the system cannot be adequately described by a two state-model.

In the light of that, an alternative approach was taken to extract exchange rates for LZ2 C335A. A linear initial rate approximation was used taking an early segment of the exchange curve ranging from 0–150 ms. Exchange rates were derived from the slope of the tangent to this segment following a standard mathematical model [26]. In practice, the increase of intensities of the cross-peaks at the start of the heteronuclear correlation experiment were taken as representative of the population changes of the two conformational states of LZ2 C335A due to exchange. The populations of each state as a function of time (*p*_1_(t) and *p*_2_(t)) together with their derivatives form a differential equation, the solution of which can be approximated by a linear function. From the resulting linear model, the exchange rate can be extracted.

In total, exchange rates were determined for 8 pairs of residues for LZ2 C335A/E342C, 4 pairs of LZ2 C335A/P301C/E342C and 5 pairs of LZ2 C335A at a temperature of 298K. Of these, those pairs with NOEs above 0.6 were selected as representative of the structured part of the protein and the average exchange rate along with standard deviations were determined. The average exchange rate for LZ2 C335A was k_ex_ = 150.3 ± 12.6 s^-1^, for LZ2 C335A/E342C k_ex_ = 8.13 ± 1.80 s^-1^ and for LZ2 C335A/P301C/E342C the average exchange rate was k_ex_ = 6.05 ± 0.97 s^-1^. These results show clearly that the introduction of N- and C-terminal disulfide bond has a significant effect on the exchange rate.

### Temperature dependency of the exchange rates

**Fig 10:**
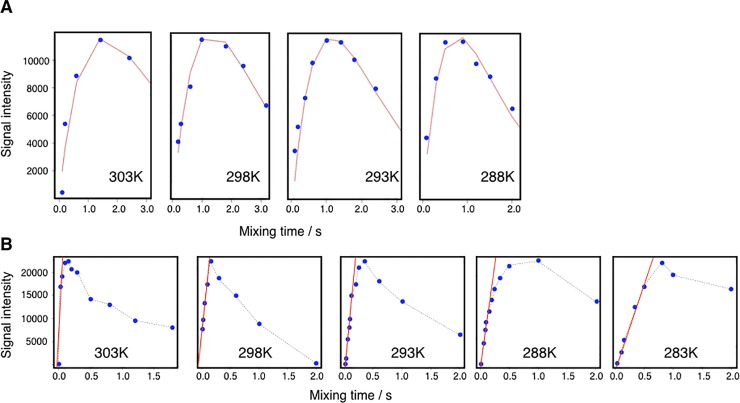
**A)** Temperature series of fitted exchange plots for E324 of LZ2 C335A/E342C from from 288K to 303K. **B)** Temperature series of fitted exchange graphs for E324 of LZ2 C335A from 283K – 3298K. Data for both were obtained at 900 MHz. Data points are shown in blue and fitted curves/lines are shown in red.

Series of heteronuclear correlation exchange experiments across a range of temperatures were recorded for all mutants. However, we were only able to get good, complete datasets for the wildtype analogue LZ2 C335A and for LZ2 C335A/E342C. The different mutants were subjected to the following temperature ranges: 283K – 308K for LZ2 C335A (at 900 MHz) and 278K -308K for LZ2 C335A/E342C (at 700 MHz and 900 MHz). These temperature ranges were chosen for the following reason: for the first series that was recorded with LZ2 C335A/E342C, the boundaries were set as far as possible to test the suitability of the experimental setup and the behavior of the sample. The range of temperatures used for the other mutants was based on the experiences made with LZ2 C335A/E342C. For each series, experiments were recorded at increments of 5K. Fig 10 shows two series of fitted exchange plots, each representing a different temperature range, put next to each other. In Fig 10A the exchange curves of E324 of LZ2 C335A/E342C, starting at 288K to the left and ending at 303K to the right are displayed. For that temperature range, exchange rates go from 4.2 s^-1^ (288K) to 9.5 s^-1^ (303K). Beneath, Fig 10B shows a sequence of exchange graphs for residue E324 of LZ2 C335A, across a temperature range of 283K – 298K. It is clearly visible that the shape of the respective curves undergoes a much more significant change in the temperature series for the WT. This is reflected by a broader distribution of exchange rates across this temperature range, which extends from 31.0 s^-1^ at 283K to 169.4 s^-1^ at 298K.

The temperature series recorded with LZ2 C335A and LZ2 C335A/E342C reveals some striking patterns. Each mutant has a closely defined temperature window within which exchange can be observed. The lowest temperature for which viable exchange data can be obtained is 283K for LZ2 C335A and is slightly shifted upwards to 288K for LZ2 C335A/E342C. Towards the top range of temperatures, the situation appears reversed. While spectra recorded at 308K yield well-defined cross-peaks and well fittable data for LZ2 C335A/E342C, respective spectra recorded at the same temperature for its counterpart LZ2 C335A are far inferior in quality and the data is hard to fit by means of linear approximation.

**Fig 11:**
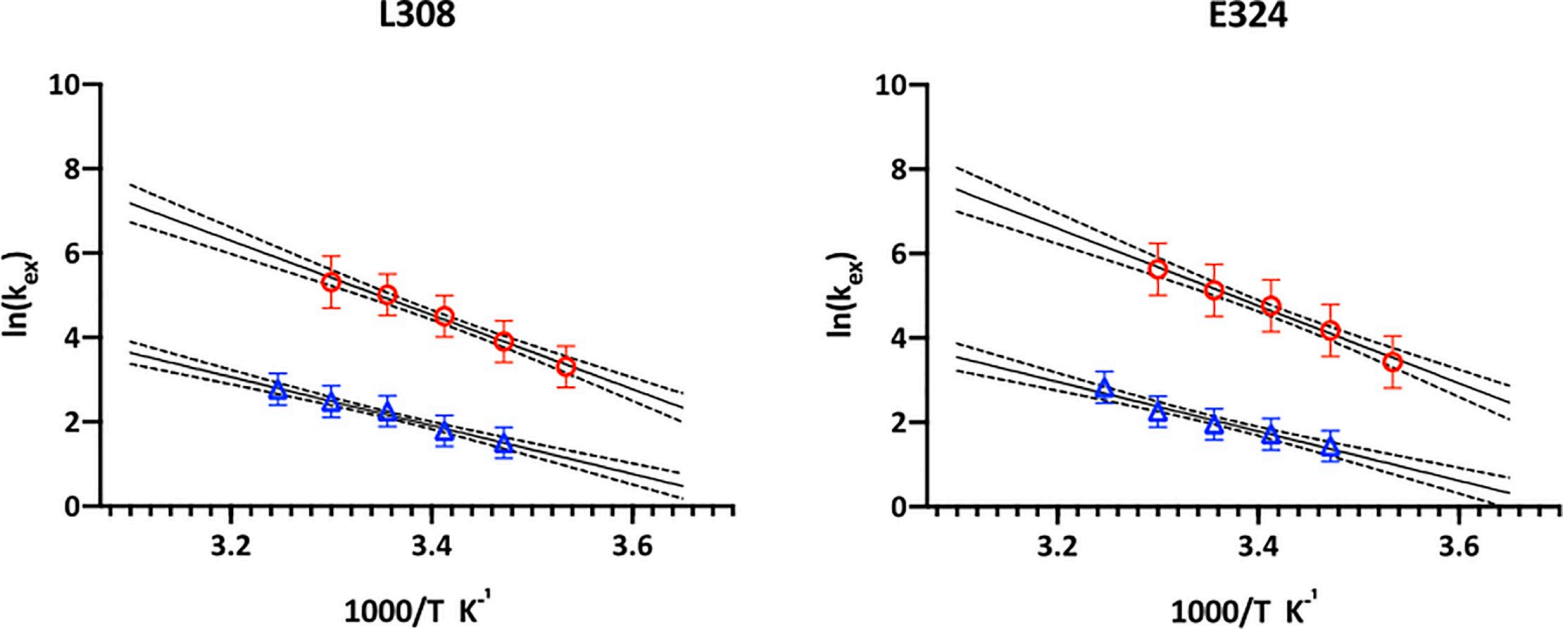
Temperature dependence of the exchange rate constants k_ex_ analysed by means of an Arrhenius plot. Data is shown for residues L308 and E324 in both LZ2 C335A (red circles) and LZ5 C335A/E342C (blue triangles). The straight line represents the linear fit, the dotted lines the 95% confidence intervals.

The temperature dependence of the exchange rates for selected residues in LZ2 C335A and LZ2 C335A/E342C was analysed in an Arrhenius plot, displayed in Fig 11. The data points matched very well a linear dependency so that a good fit was obtained. The results of the data analysis are displayed in Table 2. It was expected that the reduction of the exchange rates in the disulfide mutant would result from an *increase* of the free energy of activation. Surprisingly, introduction of a disulfide bond results in a *decrease* of ΔG^‡^ by about a factor of two. Instead, the reduction in exchange rate is caused by a change of the maximal rate constant (or pre-exponential factor) k_ex_’ by 5 to 6 orders of magnitude that far outweighs the small change of ΔG^‡^.

**Table 2:** Results of the Arrhenius analysis of the temperature dependency of the exchange rate constants for two amino acids in two LZ2 constructs.

	L308		E324	
	LZ2 C335A	LZ2 C3335A/E342C	LZ2 C335A	LZ2 C3335A/E342C
R^2^	0.937	0.925	0.922	0.896
slope	-8800 ± 631	-5741 ± 454	-9180 ± 739	-5856 ± 554
Y-intercept	34.45 ± 2.16	21.43 ± 1.53	35.97 ± 2.53	21.70 ± 1.86
ΔG^‡^ / kJ mol^-1^	73.2 ± 5.20	47.7 ± 3.77	76.3 ± 6.16	48.7 ± 4.61
k_ex_’ / s^-1^	9.15 10^14^ ± 5.73 10^13^	2.03 10^9^ ± 1.45 10^8^	4.18 10^15^ ± 2.94 10^14^	2.66 10^9^ ± 2.26 10^8^

### ^15^N Relaxation analysis

**Fig 12:**
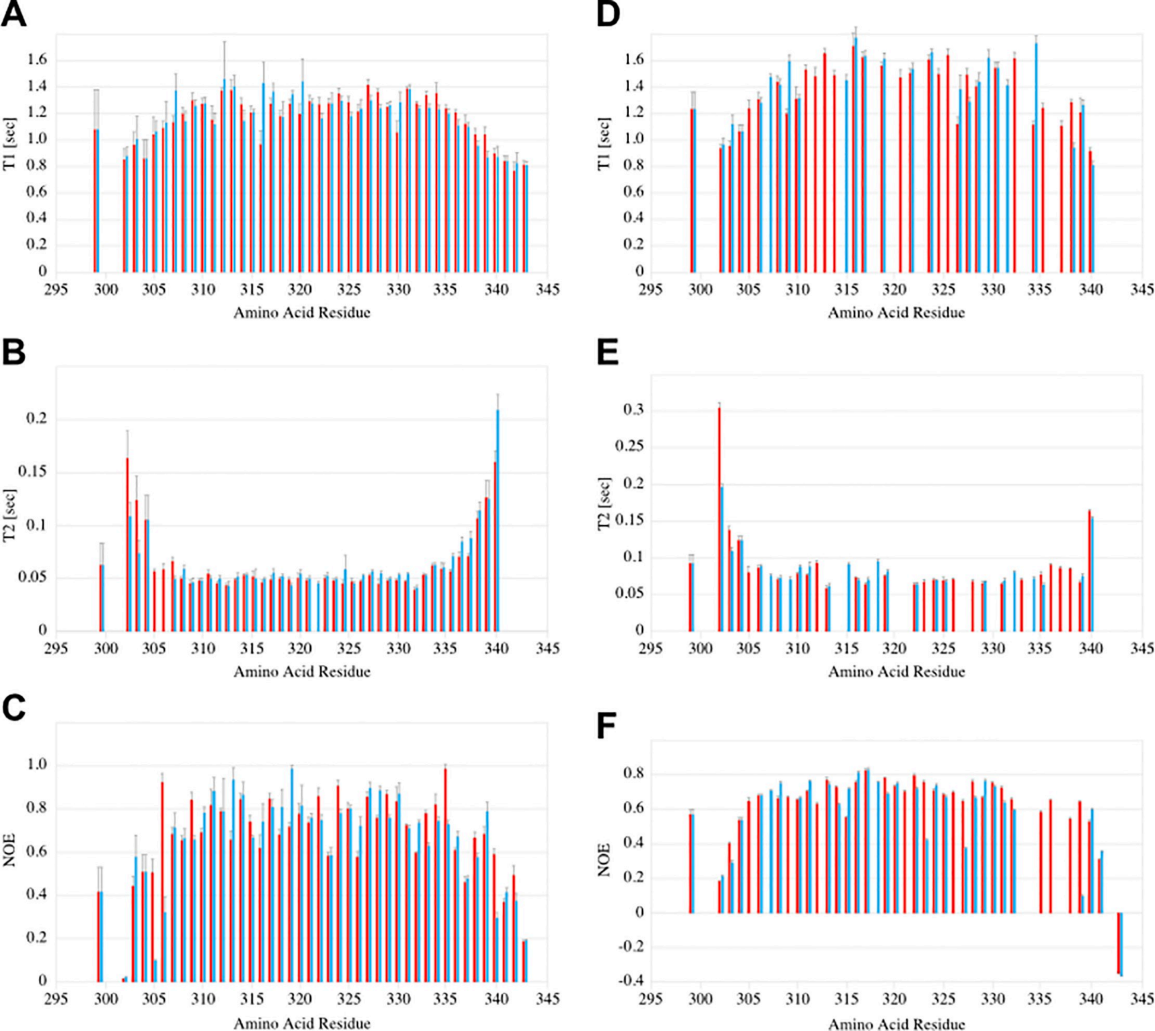
^15^N Relaxation parameters measured at 700 MHz and 298K and displayed in integrated charts which show data for conformer (A) in red and conformer (B) in blue. Data for LZ2 C335A/E342C is shown in **A)** T_1_ **B)** T_2_ **C)** ^1^H-^15^N-Heteronuclear NOEs. Data for LZ2 C335A is shown in in **D)** T_1_ **E)** T_2_ **F)** ^1^H-^15^N-Heteronuclear NOEs.

In order to confirm the extent of helical structure, potential variations between the conformers and the effect of the introduction of disulfide bonds, we measured standard ^15^N backbone relaxation data for LZ2 C335A and LZ2 C335/E342C: longitudinal relaxation times T_1_ (respectively rates R_1_), transversal relaxation times T_2_ (respectively rates R_2_) as well as ^1^H-^15^N-heteronuclear NOEs at 700 MHz. For LZ2 C335A/E342C the data are shown in Fig 12. Apart from the crowded central region, resolution of the spectra was very good. Based on the availability of a full backbone assignment of LZ2 C335A/E342C, relaxation data could be obtained for 94 of 102 residues in total. The only residues missing (G297, A298, A300 and P301) are positioned at the N-terminus.

All three relaxation parameters show little variation over the length of the structured portion of the coiled coil and are in good agreement with relaxation data previously obtained for other coiled coils [27,28]. This pattern matches the anticipated restricted mobility of amide groups residing in the coiled coil and the greater mobility displayed by amide groups positioned at the flexible ends.

For LZ2 C335A relaxation data could be determined for fewer residues than with LZ2 C335A/E342C (Fig 12). This was mainly owed to the fact that no complete backbone assignment was available and increased line broadening caused poorer resolution. T_1_ data could be obtained for 58 of 94 residues in total (32 residue of conformation A and 26 residues of conformation B). T_2_ data could be obtained for 53 of 94 residues in total (28 residues of conformation A and 25 residues of conformation B). The distribution of relaxation values across the rigid coiled coil and the flexible ends show the same trend as with LZ2 C335A/E342C, however the homogeneity of T_2_ in the coiled coil region is less pronounced.

The availability of relaxation data for LZ2 C335A and LZ2 C335A/E342C makes it possible to analyze the impact of the disulfide bond on local backbone dynamics on a fast timescale. A comparison of the ^1^H-^15^N-hetero-NOEs of L340, A341 and D343A provides a nuanced picture. While the ^1^H-^15^N hetero-NOEs of L340(A), A341(A) and A341(B) are very similar in both mutants, a NOE of 0.56 for L340(B) in LZ2 C335A suggests less flexibility than a NOE of 0.30 for its counterpart in LZ2 C335A/E342C. The most notable difference however can be detected for the C-terminal residue D343. With LZ2 C335A, the NOEs for this residue turn negative in both conformations while the NOEs of their counterparts in LZ2 C335A/E342C are around 0.18. This suggests that the introduction of the disulfide bond leads to a marked increase of the rigidity of the C-terminus. With respect to T_1_ and T_2_, A341 shows a similar pattern in both mutants. T_2_ values show a significant rise with respect to T_2_ values displayed by coiled coil residues, while T_1_ values display a significant drop. For both conformers of L340 however, the pattern of T_1_ and T_2_ values for LZ2 C335A differs from that for LZ2 C335A/E342C. In LZ2 C335A/E342C, T2 values of L340 are around 40% above the average T_2_ values of residues forming the coiled coil, suggesting an increased level of flexibility. This marks a stark contrast to LZ2 C335A/E342C, where T_2_ values of L340 are on the same level as those of the coiled coil residues. Taken together, this gives a nuanced picture of the impact of the disulfide bond on local backbone dynamics on a fast timescale. While the disulfide bond makes the transition from rigid coiled coil residues to flexible C-terminal residues steeper, it provides some stabilization to the C-terminus, which is lacking in LZ2 C335A. Table 3 gives an overview of the average differences of various relaxation parameters between exchanging pairs of residues belonging to different conformations for LZ2 C335A and LZ2 C335A/E342C.

**Table 3:** Overview of the average differences of various relaxation parameters between exchanging pairs of residues for LZ2 C335A and LZ2 C335A/E342C.

Average difference	LZ2 C335A	LZ2 C335A/E342C
**ΔT1**	**0.24 [s]**	**0.082 [s]**
**ΔT2**	**0.017 [s]**	**0.013 [s]**
**Δ**^**1**^**H-**^**15**^**N-hetero-NOE**	**0.18 [unitless]**	**0.11 [unitless]**

As a general trend for all relaxation parameters and both mutants, pairs of exchanging residues tend to have similar values, suggesting a similar structure for both conformers. However, a number of residues make an exception and display marked differences. Interestingly, for some of these residues, a significant difference in one relaxation parameter is accompanied by similar differences in other relaxation parameters. Examples for this pattern are L306 and R316 where significant differences in ^1^H-^15^N-NOEs are accompanied by a gap between T_1_ values. D343 shows an opposite pattern, where a marked difference in T_2_-values is accompanied closely by T1 and ^1^H-^15^N-NOEs.

## Discussion

The ability of a protein to execute a specific function is closely linked to a particular three-dimensional structure it adopts. Vitally important processes such as ligand recognition, enzymatic activity, cargo transport and signal transmission crucially depend on the structure of involved protein regions. Although significant progress has been made in understanding how different conformations of a particular protein are linked to specific functions, very little is known about the process of interconverting between these states. NMR is ideally suitable to analyze protein dynamics. In the case of the Nek2 leucine zipper, the intermediary/slow timescale, on which the conformational exchange process operates, precluded further structural and dynamic characterization of this protein.

The targeted introduction of non-native disulfide bonds represents an important tool in protein engineering and has frequently been used for a wide range of different purposes [29], to study protein folding [30,31], to investigate structural changes of particular protein regions and analyze allosteric mechanisms [32,33]. In particular, disulfide bonds have proven a useful tool in the study of coiled coils. In this context, they have been used to keep the α-helices forming a homodimeric dimeric coiled coil in register [7]. They have also been deployed as conformational probes reporting on a specific heptad arrangement of the coiled coil they had been inserted in [34].

This study uses protein disulfide engineering to specifically impact on the dynamics of the conformational exchange process without affecting the equilibrium populations. Thus it represents a logical continuation and complementation of previous investigations performed earlier by our group, which aimed at shifting the thermodynamic equilibrium of the exchange process by inserting the disulfide bond into the rigid part of the coiled coil [6]. In contrast to previous investigations, in this case the disulfide bond was placed into either of the flexible regions flanking the Nek2 leucine zipper, or both. This approach follows observations made by a number of groups, who noted that disulfide bonds could better be accommodated in flexible regions than in rigid protein parts [29,35,36]. A possible explanation for this might be that these regions are better suited to make the necessary backbone adjustments to prevent structural strain.

We were able to express, purify and characterize a number of such ‘kinetic’ disulfide-containing mutants of the LZ2 construct of Nek2 kinase. SDS-PAGE, NMR and CD spectroscopy showed clearly that the disulfides formed under controlled, non-reducing conditions (S1 Fig). A combined analysis of helical content from CD, secondary chemical shifts from the NMR assignment and ^15^N relaxation data (Figs 3, 6 and 12) agree very well that the LZ2 construct with or without disulfide bond is folded in a α-helical conformation from S304 to R339 in all constructs that could be comprehensively analysed. More detailed NMR experiments with all disulfide-bearing mutants show clear signs indicative of a shift in the exchange kinetics, such as sharper linewidths (Fig 8) and elimination of coherence transfer leakage (Fig 4). This allowed us to obtain a complete backbone assignment for LZ2 C335A/E342C, the mutant carrying a disulfide bond at the C-terminus (Figs 4 & 5).

Chemical shifts report on a large number of global and local factors related to the magnetic environment of a particular nucleus. Chemical shifts for a range of nuclei were compared between the 2 conformations in order to identify discrepancies pointing to structural differences between the two conformers. In general, spins of the flexible ends displayed larger chemical shift differences between the two conformations. This pattern emerges very clearly for C-terminal residues. The most striking difference between the two conformations is provided by V336. A significant distance separates the peaks corresponding to these residues in the ^15^N-HSQC, which is reflected by a Δ^15^N of 3.7 ppm. As shown in Fig 8 V336 is the only residue for which the α-helix is interrupted in one conformation. From a structural perspective, this is the most obvious difference between the two conformers of LZ2 C35A/E342C.

The effect of the disulfide bond on the exchange dynamics was comprehensively analyzed by means of heteronuclear exchange experiments. Data obtained for three of the mutants (LZ2 C335A, LZ2 C335A/E342C and LZ2 C335A/P301C/E342C) showed that disulfide bonds strategically placed into specific positions of the Nek2 leucine zipper resulted in a significant reduction of the exchange rate. Subsequent NMR experiments with the best-behaved mutant, LZ2 C335A/E342C demonstrated a substantial reduction in linewidths and the elimination of coherence transfer leakage in 3D experiments. These are observations that are in excellent agreement with a reduced frequency of exchange. Although the introduction of an additional disulfide bond at the N-terminus (LZ2 C335A/P301C/E342C) slowed down the exchange dynamics further, its impact was much smaller compared to the initial C-terminal disulfide bond. Unfortunately, samples of LZ2 C335A/P301C aggregated beyond a certain concentration, which was required to obtain extensive exchange data at multiple temperatures. The exchange rate measured in this work for LZ2 C335A at 298 K are unexpectedly much larger than those measured for the slightly shorter construct LZ5 used in our previous work (6). At this point and without accurate structures of both conformers and the transition state it is difficult to speculate about this large difference which remains to be resolved.

The analysis of the temperature dependence of the exchange rates of various constructs gave high quality data (Figs 10 and 11, Table 2)–albeit only for two residues–resulting in a good fit and very similar results for both residues (L308 and E324). The obtained activation energies match well those observed for analogue dynamic processes in the α-helical region of the prokaryotic Nitrogen Regulatory Protein C (NtrC) [37] and conformational exchange near the chromophore in GFP [38]. Also the pre-exponential factors k_ex_’ match values observed for dynamic processes in proteins [39]. However, the ΔG^‡^ values for LZ2 C335A are about twice as high than for LZ2 C335A/E342C. This difference in activation energy would actually be expected to lead to *faster* exchange in the disulfide mutant which is clearly not what we observe. It is therefore necessary to consider the second parameter that we have extracted from the temperature dependence of the exchange rate constant, the pre-exponential factor k_ex_’. This parameter shows a huge difference as a function of the presence of disulfide bonds–a change of 4–5 orders of magnitude. This substantial difference is clearly dominating the comparatively small change in ΔG^‡^ and thus explains our observations.

The process of determining exchange rates encompasses a number of potential sources of errors, such as the temperature calibration within the spectrometer, intensity measurement of cross-peaks and fitting of data among others. As previously described, this can lead to significant errors of 25% and above [40]. However, even larger errors in exchange rates only mildly affect the calculated activation energies due to the logarithmic relation of the two. Thus, only very large changes in exchange rates have a marked impact on activation energies [41,42]. Furthermore, the fact that exchange rate constants could only be measured over a small temperature range which is then extrapolated to infinite temperature adds a further level of uncertainty to the quantitative analysis.

In addition, for the analysis of the exchange dynamics of the Nek2 LZ we have only good data for two residues across the two mutants which further limits the conclusions we can draw. Therefore, while the detailed numbers might not be the most precise it is quite clear from our analysis that the main contribution to the kinetic effect of the disulfide bridges does not arise from an increase in ΔG^‡^ but from a significant reduction of the pre-exponential factor or maximal possible exchange rate constant k_ex_’. How can this be rationalized for the conformational exchange of the Nek2 leucine zipper? The maximal exchange rate k_ex_’ indicates the number of collisions with the required geometry necessary for a reaction to occur [11]. The activation energy ΔG^‡^ is a result of the need of a system to adapt a configuration that is conducive for the reaction to occur, e.g. the deformation of bonds in chemical reactions. This makes the exponential factor in Eq (1) a proportionality factor that determines how much of the maximal possible conducive collisions described by k_ex_’ do indeed lead to a reaction. The halving of ΔG^‡^ by the addition of a disulfide bridge could therefore be explained by its ability of holding the two helices together when the conformational change occurs. This could indicate that there is a bit of an effort to reorient the two helices which is supported by a covalent linkage between the individual monomers. In contrast, the huge drop in the maximum attainable rate constant k_ex_’ indicates that the presence of a disulfide bond drastically reduces the chances that the leucine zipper can adopt a conformation that is conducive to a conformational interconversion.

### Outlook

Disulfide engineering is becoming increasingly important and the scope of applications for which it is used is widening. There is also a growing appreciation of the role interconversion dynamics play for the capacity of proteins to carry out multiple functions. Our study puts forward a new purpose to the existing scope of applications of disulfide engineering. It could therefore be applied to a range of molecular systems which display exchange on a slow to intermediary timescale, in particular to those that have proven intractable for analysis by X-ray crystallography and NMR so far. Furthermore, the Nek2 leucine zipper could serve as a model system to get a deeper understanding of the dynamics of coiled coils. This fundamental dimerization domain is widespread, it represents a crucially important tool for proteins to associate and is frequently used in protein engineering [43]. Thus, a deeper understanding of its dynamics could be very valuable for protein engineering, drug discovery, protein interaction studies and other disciplines.

## Supporting information

S1 Fig **A)** SDS PAGE analysis of disulfide bond formation. Samples of LZ2 mutants C335A/P301C and C335A/E34C are shown in reduced and oxidized states. **B)** HNCACB-strips belonging to residues C342(A) and C342(B) of LZ2 C335A/E342C showing β-carbon-signals (C_β_) with chemical shifts characteristic of cysteine residues engaged in a disulfide bond. **C)** CD melting experiments in the absence and presence of DTT of the disulfide engineered constructs used in this work. The green dotted line for the mutant C335A/E342C is a repeat experiment to test the reversibility of thermal unfolding. **D)** Analysis of peak volume ratios of exchanging pairs of peaks for the same amino acid. Ratios of peak volumes for well resolved peaks representing the two conformers of 12 residues were calculated and plotted (blue triangles) with the calculation of the mean (horizontal bar) and the 95% confidence interval (grey box). Note that LZ2 C335A has a much broader distribution of ratios despite having a virtually identical average as LZ2 C335A/E342C, presumably due to the broader lines, overlap and generally poorer quality of the spectrum. **E)** Spectra of mutant LZ2 C335A/E342C in reduced (green) and oxidized (yellow) states. Peak positions for most residues in the coiled-coil core are essentially unaffected by the change in redox status suggesting that structure is little affected while the linewidths have increased
(TIFF)

S2 Fig **A)** Representative example of a fitted plot of cross-peak intensity as a function of mixing time for A341 of LZ2 C335A. For this fit, the same mathematical model was used as for the fits shown in Fig 9B and 9C (data obtained at 298K) **B)** Illustration of the alternative approach taken to extract exchange rates for LZ2 C335A. A segment of the (intensity as a function of mixing time) curve covering the signal ascent from 0 to 150 ms was approximated in a linear way and the calculated slope of the tangent yielded the exchange rate. The data was obtained at 298K.
(TIFF)

S1 Table **A)** Assignments for conformation (A) of LZ2 C335A/E342C **B)** Assignments for conformation (B) of LZ2 C335A/E342
(TIFF)
