# Supplementary figures and images for: Controlling the dynamics of the Nek2 leucine zipper by engineering of “kinetic” disulphide bonds
Source: PLoS One. 2019 Feb 1;14(2):e0210352. doi: 10.1371/journal.pone.0210352 (PMC6358272; doi:10.1371/journal.pone.0210352)

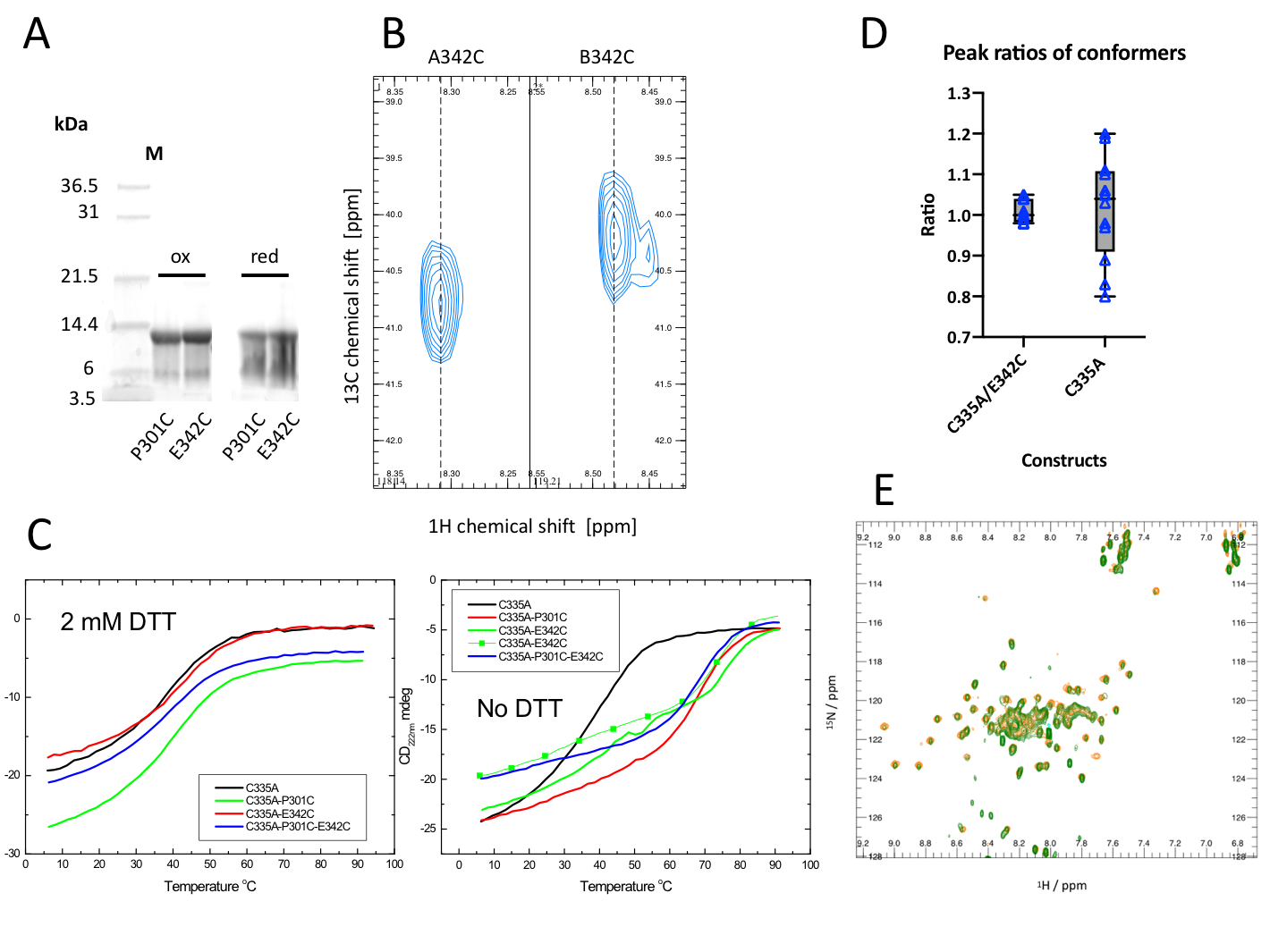

Supplement: S1 Fig — A) SDS PAGE analysis of disulfide bond formation. Samples of LZ2 mutants C335A/P301C and C335A/E34C are shown in reduced and oxidized states. B) HNCACB-strips belonging to residues C342(A) and C342(B) of LZ2 C335A/E342C showing β-carbon-signals (Cβ) with chemical shifts characteristic of cysteine residues engaged in a disulfide bond. C) CD melting experiments in the absence and presence of DTT of the disulfide engineered constructs used in this work. The green dotted line for the mutant C335A/E342C is a repeat experiment to test the reversibility of thermal unfolding. D) Analysis of peak volume ratios of exchanging pairs of peaks for the same amino acid. Ratios of peak volumes for well resolved peaks representing the two conformers of 12 residues were calculated and plotted (blue triangles) with the calculation of the mean (horizontal bar) and the 95% confidence interval (grey box). Note that LZ2 C335A has a much broader distribution of ratios despite having a virtually identical average as LZ2 C335A/E342C, presumably due to the broader lines, overlap and generally poorer quality of the spectrum. E) Spectra of mutant LZ2 C335A/E342C in reduced (green) and oxidized (yellow) states. Peak positions for most residues in the coiled-coil core are essentially unaffected by the change in redox status suggesting that structure is little affected while the linewidths have increased (TIFF) [file pone.0210352.s001.tiff]

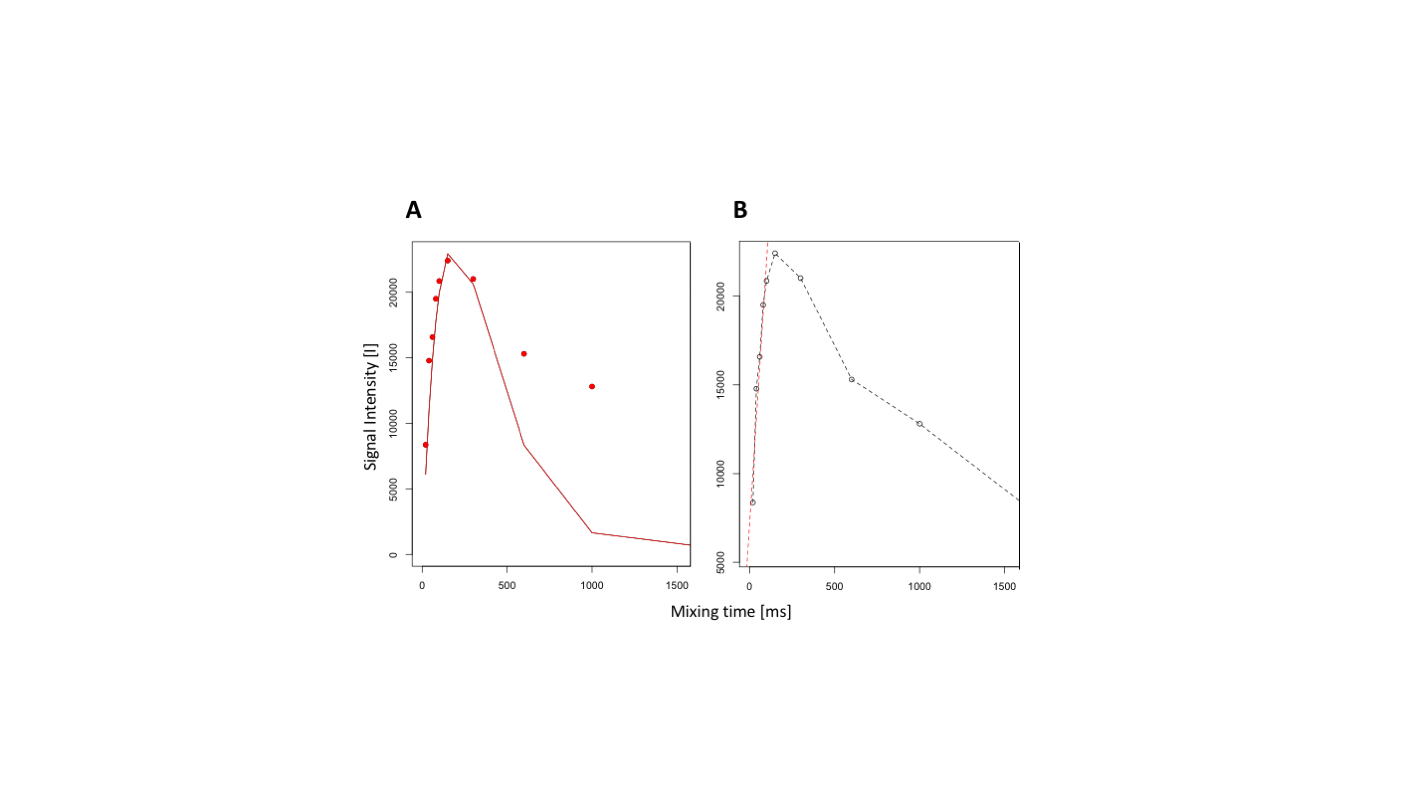

Supplement: S2 Fig — A) Representative example of a fitted plot of cross-peak intensity as a function of mixing time for A341 of LZ2 C335A. For this fit, the same mathematical model was used as for the fits shown in Fig 9B and 9C (data obtained at 298K) B) Illustration of the alternative approach taken to extract exchange rates for LZ2 C335A. A segment of the (intensity as a function of mixing time) curve covering the signal ascent from 0 to 150 ms was approximated in a linear way and the calculated slope of the tangent yielded the exchange rate. The data was obtained at 298K. (TIFF) [file pone.0210352.s002.tiff]

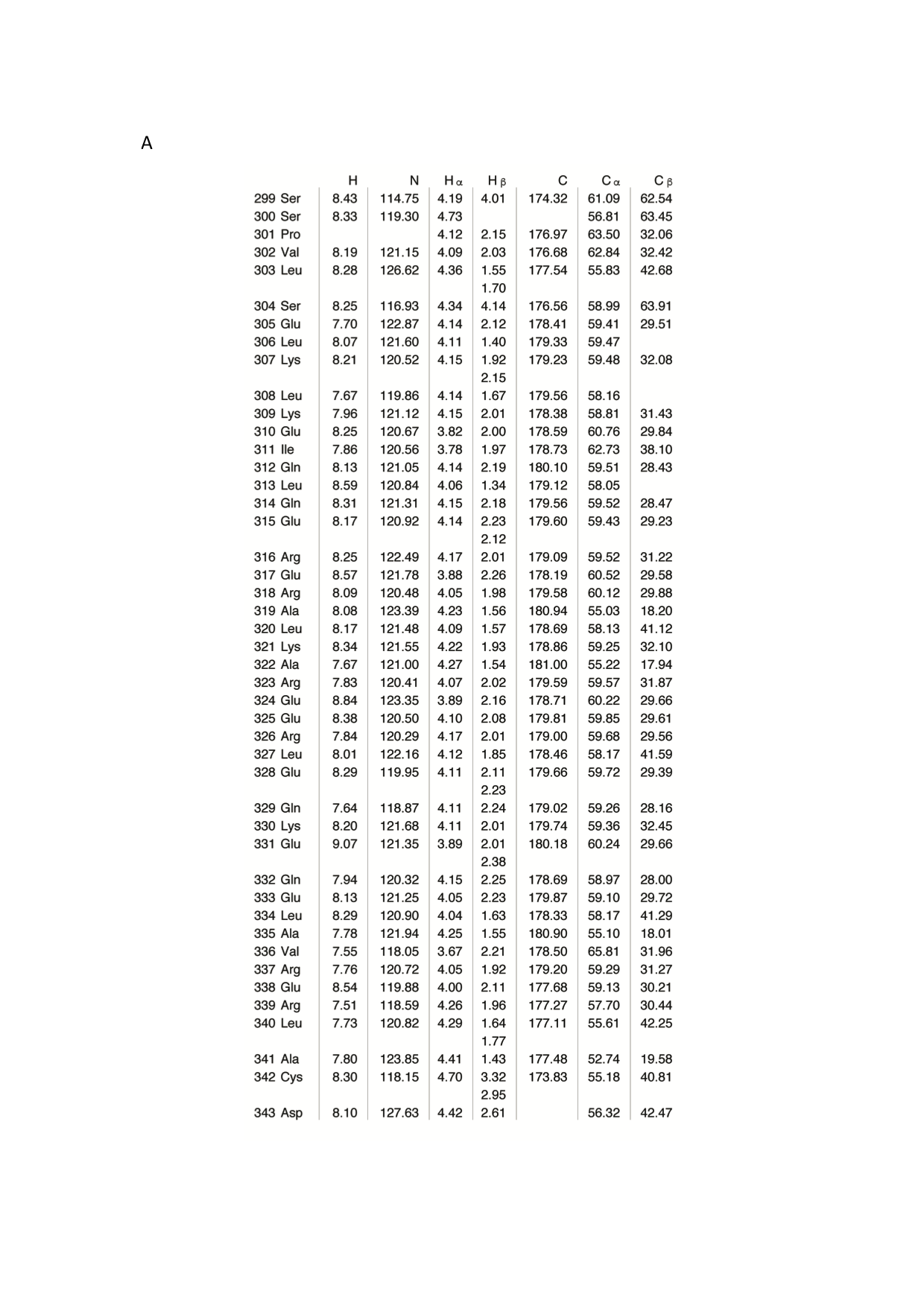

Supplement: S1 Table — A) Assignments for conformation (A) of LZ2 C335A/E342C B) Assignments for conformation (B) of LZ2 C335A/E342 (TIFF) [file pone.0210352.s003.tiff]
